# Edge-illumination spectral phase-contrast tomography

**DOI:** 10.1088/1361-6560/ad3328

**Published:** 2024-04-03

**Authors:** Luca Brombal, Fulvia Arfelli, Francesco Brun, Vittorio Di Trapani, Marco Endrizzi, Ralf H Menk, Paola Perion, Luigi Rigon, Mara Saccomano, Giuliana Tromba, Alessandro Olivo

**Affiliations:** 1 Department of Physics, University of Trieste, Via A. Valerio 2, I-34127 Trieste, Italy; 2 INFN Division of Trieste, Via A. Valerio 2, I-34127 Trieste, Italy; 3 Department of Engineering and Architecture, University of Trieste, Via A. Valerio 10, I-34127 Trieste, Italy; 4 Department of Medical Physics and Biomedical Engineering, University College London, Gower Street, GWC1E 6BT, London, United Kingdom; 5 Elettra-Sincrotrone Trieste S.C.p.A, I-34149 Basovizza Trieste, Italy; 6 Department of Computer and Electrical Engineering, Midsweden University, Holmgatan 10, Sundsvall, Sweden; 7 Helmholtz Zentrum München, Helmholtz Pioneer Campus, Ingolstädter Landstraße 1, D-85764 Neuherberg, Germany

**Keywords:** x-ray phase-contrast imaging, spectral imaging, edge illumination, photon-counting detector, x-ray tomography

## Abstract

Following the rapid, but independent, diffusion of x-ray spectral and phase-contrast systems, this work demonstrates the first combination of spectral and phase-contrast computed tomography (CT) obtained by using the edge-illumination technique and a CdTe small-pixel (62 *μ*m) spectral detector. A theoretical model is introduced, starting from a standard attenuation-based spectral decomposition and leading to spectral phase-contrast material decomposition. Each step of the model is followed by quantification of accuracy and sensitivity on experimental data of a test phantom containing different solutions with known concentrations. An example of a micro CT application (20 *μ*m voxel size) on an iodine-perfused *ex vivo* murine model is reported. The work demonstrates that spectral-phase contrast combines the advantages of spectral imaging, i.e. high-*Z* material discrimination capability, and phase-contrast imaging, i.e. soft tissue sensitivity, yielding simultaneously mass density maps of water, calcium, and iodine with an accuracy of 1.1%, 3.5%, and 1.9% (root mean square errors), respectively. Results also show a 9-fold increase in the signal-to-noise ratio of the water channel when compared to standard spectral decomposition. The application to the murine model revealed the potential of the technique in the simultaneous 3D visualization of soft tissue, bone, and vasculature. While being implemented by using a broad spectrum (pink beam) at a synchrotron radiation facility (Elettra, Trieste, Italy), the proposed experimental setup can be readily translated to compact laboratory systems including conventional x-ray tubes.

## 1. Introduction

The field of x-ray imaging is evolving due to the diffusion of spectral and phase-contrast techniques. In the context of clinical applications, the recent advent of computed tomography (CT) scanners equipped with photon-counting detectors has paved the way for detector-based spectral imaging (Willemink *et al* 2018). Compared to the previous generation of dual-energy scanners based on dual-source, voltage switching, or dual-layer sensors, new systems implementing photon-counting detectors with multiple energy thresholds bring significant hardware simplification and increased flexibility (Hsieh *et al* 2020). Additionally, photon counters have been shown to decrease image noise at low fluxes, owing to electronic noise rejection, and to improve image contrast, due to the absence of spectral weighting (Flohr *et al* 2020). At the same time, the availability of small-pixel (<100 *μ*m) photon-counting devices is impacting micro-CT (*μ*CT) systems dedicated to pre-clinical or non-clinical studies, enabling material discrimination in 3D at the micrometer scale by using polychromatic x-ray sources (Badea *et al* 2019, Brun *et al* 2020, Paakkari *et al* 2021). Spectral CT uses energy-binned images of the sample to produce material-specific 3D maps of a given quantity, typically the mass density (*ρ*) (Roessl and Proksa 2007). More in detail, the different energy dependence of the attenuation coefficient of elements with different atomic numbers (*Z*) is used to discriminate among materials, in a process known as spectral or material decomposition (Alvarez and Macovski 1976). On the other hand, since spectral CT is based on x-ray attenuation, it suffers from the low contrast produced by weakly absorbing materials, therefore hampering the possibility of discriminating and quantifying low-*Z* biological tissues. To overcome this limitation, high-*Z* contrast agents are often used to target specific anatomical or functional structures of interest. Specifically, the sharp increase in attenuation seen in correspondence with the contrast agent’s K-edge can be exploited to yield optimal material decomposition results, thus isolating the target organ (contrast image) from the remaining anatomical background (non-contrast image) (Faby *et al* 2015).

In parallel to—but independently from—the diffusion of spectral CT, x-ray phase-contrast imaging (XPCI) is becoming widely available not only at synchrotron radiation facilities (Bravin *et al* 2012), but also with compact systems based on conventional x-ray sources (Endrizzi 2018, Quenot *et al* 2022). The clinical potential of XPCI has been recently demonstrated for a number of different techniques. Promising results have been obtained for instance in the field of chest and breast imaging by using Talbot-Lau grating interferometry (GI) (Frank *et al* 2022, Rawlik *et al* 2023), in the field of intraoperative tomography by using edge illumination (EI) (Havariyoun *et al* 2019), and in the field of breast CT by using the propagation-based imaging method (Gureyev *et al* 2019, Longo *et al* 2019). An even larger number of studies based on XPCI techniques has been published in the context of pre-clinical or non-clinical research, among which it is worth mentioning recent applications in the fields of virtual histology (Massimi *et al* 2021, Polikarpov *et al* 2023) and safety inspection (Partridge *et al* 2022). Owing to the 3-order of magnitude difference between the unit decrement of the real part (*δ*) and the imaginary part (*β*) of the complex refractive index (*n*), XPCI delivers higher visibility on low-*Z* materials, such as soft tissues, with respect to attenuation or spectral imaging. Many XPCI techniques allow the extraction of both attenuation and phase-shift maps. Since phase-shift and attenuation signals have different dependencies on the physical properties of the sample (*ρ* and *Z*), the attenuation/phase duality can be used for material decomposition (Braig *et al* 2018, Buchanan *et al* 2022). On the other hand, at the energy resolution typically available in spectral imaging systems (>1 keV), the phase signal does not feature K-edge discontinuities hence being comparatively less effective when contrast media are used.

In this context, it is clear that the combination of spectral and phase-contrast techniques would bring both high visibility of low-*Z* biological tissues and effective separation of high-*Z* materials (contrast media). So far, due to the limited availability of phase-contrast systems equipped with spectral detectors, only a relatively small number of spectral phase contrast studies have been reported in the literature (Mechlem *et al* 2019, Schaff *et al* 2020, Astolfo *et al* 2023, Brombal *et al* 2023a). The most illustrative example is arguably the one provided by Ji *et al* (2020) where a GI system is coupled with a 100 *μ*m pixel size photon-counting detector. In their paper, the authors introduce a theoretical model of spectral phase contrast and demonstrate it on a test object and a simple phantom.

In this work, the first integration of spectral phase contrast in a tomographic EI setup is demonstrated. The system includes a small-pixel (62 *μ*m) spectral detector and the technique is applied to a test phantom and an *ex vivo* murine model perfused *post-mortem* with an iodine-based contrast agent. High-resolution images are obtained (down to 20 *μ*m voxel size) and quantification of soft tissue (water), bone (calcium), and contrast medium (iodine) is simultaneously performed. A step-by-step theoretical model is introduced transitioning from standard spectral material decomposition to attenuation phase-contrast duality and, finally, to spectral phase contrast. Each theoretical step is followed by imaging results where the accuracy and sensitivity of the decomposition are quantitatively compared. The acquisitions are performed at the Elettra Synchrotron facility (Trieste, Italy) by employing a filtered polychromatic beam (pink beam). As will become clear in the following, this application can be readily translated into laboratory systems based on conventional x-ray tubes.

## 2. Materials and methods

### 2.1. Spectral, attenuation/phase, and spectral/phase decomposition algorithms

To describe both spectral and phase-contrast effects, it is convenient to use an x-ray/matter interaction model where the sample is defined by the three-dimensional distribution of its complex refractive index *n* = 1 − *δ* + i*β*. Here, the complex term *β* = *hc*/(4*π* *E*) · *μ*_*a*_, where *h* is Plank’s constant, *c* is the speed of light, and *E* is the x-ray energy, is proportional to the absorption coefficient *μ*_*a*_ (Als-Nielsen and McMorrow 2011). The decrement from unity of the real part *δ* is proportional to the phase shift.

The physical information contained in conventional CT is the attenuation coefficient which, in the diagnostic radiology energy range (10–150 keV), can be written as:

\begin{eqnarray*}\mu (E,Z)=\rho \left[{f}_{{PE}}(E,Z)+{f}_{K}(E,Z)+{f}_{{CS}}(E)\right]\end{eqnarray*}

where *f*_*PE*_(*E*, *Z*), *f*_*K*_(*E*, *Z*) are the energy and atomic number dependent cross sections (per unit of mass) accounting for the photo-electric absorption, i.e. *μ*_*a*_ = *ρ*(*f*_*PE*_ + *f*_*K*_), far from and across K-edge absorption energies, respectively; *f*_*CS*_(*E*) is the energy-dependent, but atomic number independent, Compton scattering cross section calculated according to the Klein-Nishina theory (Evans 1955, Sukovle and Clinthorne 1999, Schlomka *et al* 2008). By probing attenuation at different energies, material discrimination is allowed owing to the material-specific energy dependence of the cross sections. The previous equation explicitly contains the proportionality to the mass density (*ρ*) as cross sections are typically tabulated per unit of mass.

On the other hand, the phase term of the refractive index, which is measured in XPCI CT, has simpler energy and material dependencies:

\begin{eqnarray*}\delta (E)=\rho \displaystyle \frac{Z}{A}\displaystyle \frac{{r}_{0}{N}_{A}{h}^{2}{c}^{2}}{2\pi {E}^{2}}\simeq \rho \displaystyle \frac{{r}_{0}{N}_{A}{h}^{2}{c}^{2}}{4\pi {E}^{2}}\end{eqnarray*}

where *r*_0_ is the classical electron radius, *N*_*A*_ is the Avogadro number, and *A* is the mass number (Als-Nielsen and McMorrow 2011). Under the approximation *Z*/*A* ≃ 1/2, which holds true for most of the biological tissues, *δ* is proportional to the mass density and independent of the material atomic number. Interestingly, the latter feature implies that at a fixed energy *δ* is proportional to the Compton scattering cross-section *f*_*CS*_.

The quantification of specific materials in an x-ray dataset is performed through material decomposition algorithms, where two or more linearly independent input images are written as a linear combination of basis material output images. The linear combination coefficients are related to some physical property of the basis material and they are usually computed through matrix inversion. Specifically, in CT, the material decomposition problem can be formulated to yield 3D maps of the mass density. The following sections describe three material decomposition algorithms representing three different x-ray imaging approaches relying on (i) a standard spectral setup based on an energy-resolving detector, (ii) a standard phase-contrast system without spectral information, and (iii) a spectral phase-contrast setup including an energy-resolving detector. All algorithms are based on matrix inversion but vary in terms of their input data. Standard spectral material decomposition takes as input two attenuation channels at different energies. Attenuation/phase decomposition is based on a single attenuation channel (non-spectral) and a phase channel. Finally, spectral/phase decomposition is based on two spectral attenuation channels and one phase channel.

#### 2.1.1. Spectral material decomposition

When dealing with attenuation-based spectral decomposition, each attenuation image is written as a linear combination of mass attenuation coefficients of given basis materials (i.e. the decomposition materials):

\begin{eqnarray*}\mu =\displaystyle \sum _{i}{\rho }_{i}{\left(\displaystyle \frac{\mu }{\rho }\right)}_{i}\end{eqnarray*}

where the weights correspond to the mass density, the index *i* runs over the number of chosen basis materials, and the equivalence has to be intended on a voxel-by-voxel basis. In the previous equation, the dependence on *E* has been dropped since, as it will be clear in the following section, *μ*/*ρ* values are typically given as mean values integrated over the energy spectrum.

Considering the case of a spectral imaging system producing attenuation images over two energy bins, which is the most common scenario when using small-pixel chromatic detectors or dual-energy systems, equation (3) can be extended to a system of linear equations

\begin{eqnarray*}\left[\begin{array}{c}{\mu }^{L}\\ {\mu }^{H}\end{array}\right]=\left[\begin{array}{cc}{\left(\displaystyle \frac{\mu }{\rho }\right)}_{1}^{L} &amp; {\left(\displaystyle \frac{\mu }{\rho }\right)}_{2}^{L}\\ {\left(\displaystyle \frac{\mu }{\rho }\right)}_{1}^{H} &amp; {\left(\displaystyle \frac{\mu }{\rho }\right)}_{2}^{H}\end{array}\right]\left[\begin{array}{c}{\rho }_{1}\\ {\rho }_{2}\end{array}\right]\end{eqnarray*}

the superscripts *L* and *H* refer to the low and high energy bins, respectively, while the subscripts 1 and 2 indicate two decomposition materials. At this point, material-decomposed density maps *ρ*_1,2_ can be generated by inverting the linear system, i.e. inverting the decomposition matrix. Unless other assumptions are made, as in the case of mass fraction decomposition (Liu *et al* 2009), the availability of 2 energy bins limits the decomposition to 2 materials.

It should also be mentioned that, while this formalism can be in principle applied to any pair of decomposition materials, the presence of image noise requires two basis materials with rather different energy dependencies (i.e. appreciably different atomic numbers) to avoid major noise amplification in the matrix inversion (Di Trapani *et al* 2022). For this reason, spectral decomposition is particularly suited to quantify and separate, for instance, high-*Z* contrast media from the soft tissue background. In this specific case, the optimal choice is to maximize the difference in the energy dependence of decomposition materials by selecting the energy bins above and below the K-edge energy of the contrast medium.

#### 2.1.2. Attenuation/phase material decomposition

Most XPCI CT techniques give as output both attenuation and phase maps. To apply the same formalism used for the spectral case and take into account equation (2), the phase image is written as a linear combination of *δ* per unit of mass of a given set of materials, where the weights are given by their mass densities:

\begin{eqnarray*}\delta =\displaystyle \sum _{i}{\rho }_{i}{\left(\displaystyle \frac{\delta }{\rho }\right)}_{i}\end{eqnarray*}

It is worth noting that the linear combination coefficients (*ρ*_*i*_) in the previous equation are the same as in equation (3). Equation (5) is formally equivalent to the attenuation-based case, hence making the entire decomposition problem equivalent to the one of the previous section:

\begin{eqnarray*}\left[\begin{array}{c}\mu \\ \delta \end{array}\right]=\left[\begin{array}{cc}{\left(\displaystyle \frac{\mu }{\rho }\right)}_{1} &amp; {\left(\displaystyle \frac{\mu }{\rho }\right)}_{2}\\ {\left(\displaystyle \frac{\delta }{\rho }\right)}_{1} &amp; {\left(\displaystyle \frac{\delta }{\rho }\right)}_{2}\end{array}\right]\left[\begin{array}{c}{\rho }_{1}\\ {\rho }_{2}\end{array}\right]\end{eqnarray*}

Here, instead of low-energy and high-energy attenuation bins, single energy bin attenuation and phase images are used as input, while tabulated mass attenuation and phase coefficients are used in the decomposition matrix. Differently from the spectral case, the attenuation/phase decomposition allows for the choice of basis materials with rather similar attenuation properties. In fact, while the phase channel depends only on the mass density (see equation (2)), attenuation is in general strongly dependent on *Z*, making the two decomposition bases independent, as required in the matrix inversion process. For this reason, many examples exist in literature where attenuation/phase decomposition has been used to separate and quantify e.g., plastic materials, soft tissues, and bones (Braig *et al* 2018, Navarrete-León *et al* 2023). Conversely, attenuation/phase does not provide specific advantages in distinguishing and quantifying K-edge contrast elements as input images are integrated over the whole energy spectrum.

#### 2.1.3. Spectral/phase material decomposition

Having introduced spectral and attenuation/phase decompositions individually, the extension to the case where both are combined is straightforward. Specifically, the attenuation/phase system in equation (6) can be modified by replacing the single-attenuation channel with the low and high-energy attenuation bins. Hence, the spectral-phase decomposition system becomes:

\begin{eqnarray*}\left[\begin{array}{c}{\mu }^{L}\\ {\mu }^{H}\\ \delta \end{array}\right]=\left[\begin{array}{ccc}{\left(\displaystyle \frac{\mu }{\rho }\right)}_{1}^{L} &amp; {\left(\displaystyle \frac{\mu }{\rho }\right)}_{2}^{L} &amp; {\left(\displaystyle \frac{\mu }{\rho }\right)}_{3}^{L}\\ {\left(\displaystyle \frac{\mu }{\rho }\right)}_{1}^{H} &amp; {\left(\displaystyle \frac{\mu }{\rho }\right)}_{2}^{H} &amp; {\left(\displaystyle \frac{\mu }{\rho }\right)}_{3}^{H}\\ {\left(\displaystyle \frac{\delta }{\rho }\right)}_{1} &amp; {\left(\displaystyle \frac{\delta }{\rho }\right)}_{2} &amp; {\left(\displaystyle \frac{\delta }{\rho }\right)}_{3}\end{array}\right]\left[\begin{array}{c}{\rho }_{1}\\ {\rho }_{2}\\ {\rho }_{3}\end{array}\right]\end{eqnarray*}

In this case, the availability of three input images allows for the introduction of a third decomposition material. It is worth noting that the energy dependence of *δ* is the same for all materials, as shown in equation (2), hence the addition of phase energy bins is equivalent to adding linearly dependent rows in the decomposition matrix.

**Figure 1.**
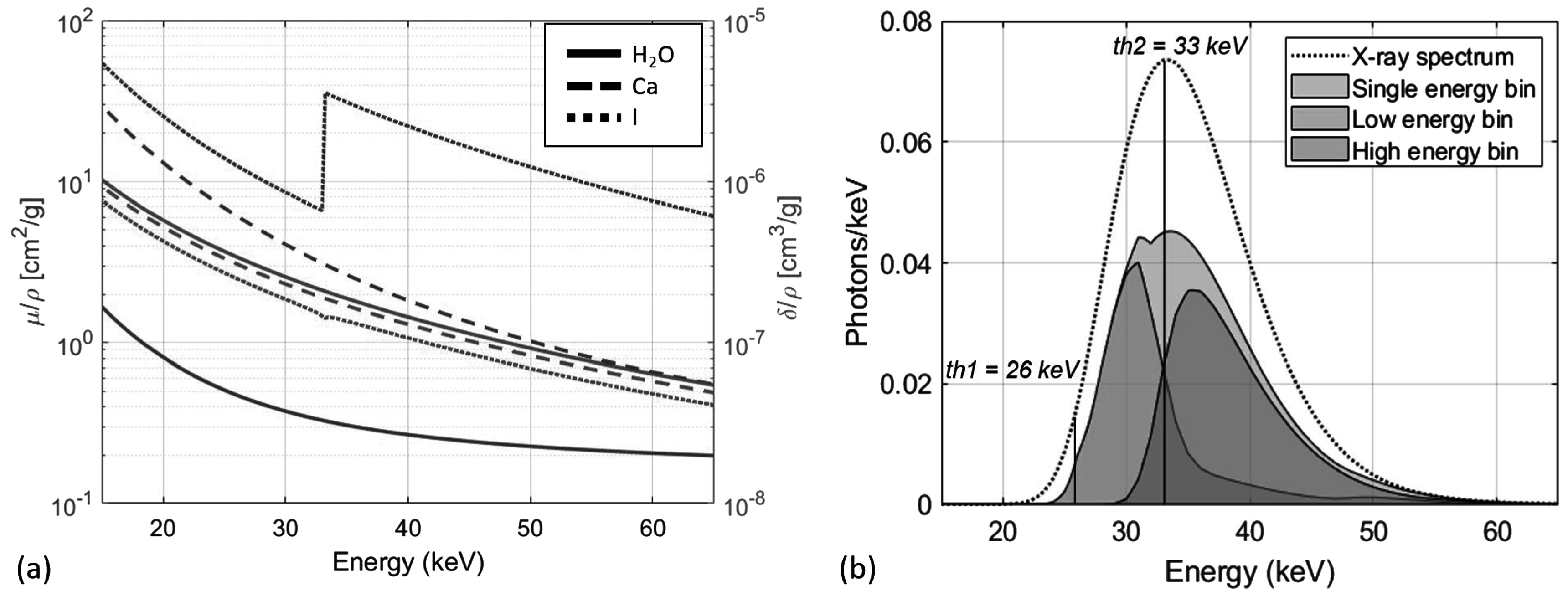
(a) Mass attenuation (blue) and phase (orange) coefficients as a function of energy of water, calcium, and iodine. (b) X-ray spectrum used for acquisition (dotted line) and energy spectral response of the system (area-filled curves).

The linear system in equation (7) combines the advantages of spectral and phase-contrast imaging for the task of material decomposition. Specifically, considering the example of a biological sample perfused *post-mortem* with a high-*Z* contrast medium, spectral information would mainly contribute to the quantification of a high-*Z* contrast medium, whereas the attenuation/phase duality would allow the separation of biological tissue components. Figure 1(a) shows the mass attenuation and phase coefficients as a function of energy for the basis materials used in this work, namely water, calcium, and iodine.

### 2.2. Edge illumination

**Figure 2.**
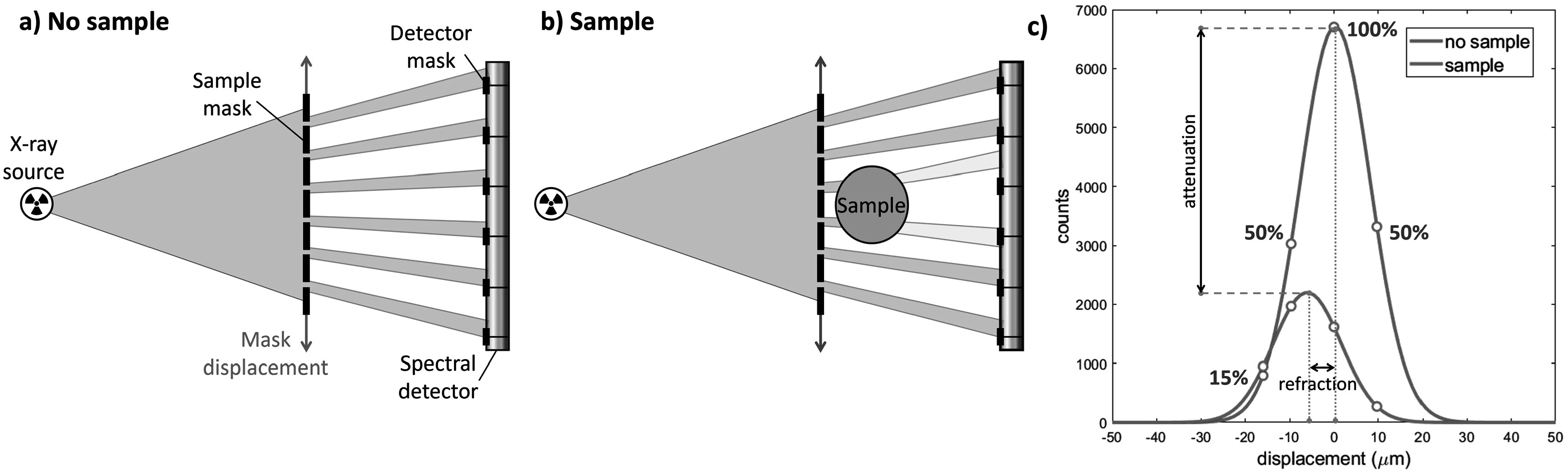
Sketch of an edge illumination setup without (a) and with (b) the sample. In (c), the illumination curves obtained without (blue line) and with (orange line) sample are shown. In the experiment, the curve was sampled the four points shown in the plots.

In this paper, phase-contrast imaging is performed by using the EI technique (Olivo and Speller 2007). This method requires the introduction of two absorbing masks, placed upstream (sample mask) and downstream (detector mask) of the sample, respectively (figure 2(a)). The sample mask shapes the beam into an array of non-interfering beamlets which reach the detector after traversing the sample. Here, insensitive regions between adjacent pixels are created by the detector mask. The two masks are geometrically equivalent, only scaled by a magnification factor. The displacement of the sample mask by a specific number of steps with respect to the detector mask, which is kept fixed, creates the illumination curve (figure 2(c)). The illumination curve is typically modeled as a Gaussian function, whose parameters are estimated from a fit performed on a minimum of three sample points (Endrizzi *et al* 2014). A reference illumination curve is obtained, for each pixel, with no sample (figure 2(a) and blue curve in (c)). When the sample is introduced, the illumination curve is dampened (attenuation) and displaced laterally (refraction) (figure 2(b) and orange curve in (c)). The measurement of these two contributions allows for the retrieval of independent attenuation and differential phase images. The latter is subsequently integrated and gives access to the sample-induced phase shift, i.e. the projection of *δ* along the sample’s thickness.

### 2.3. Spectral characterization of the system

The determination of the coefficients of the basis materials in the decomposition matrices requires a thorough spectral characterization of the imaging system, both in terms of detector energy response and input x-ray spectrum. Specifically, the spectral response of the CdTe chromatic detector used in this work (Pixirad-PixieIII), has been obtained via threshold scans with monochromatic radiation in the range 26–50 keV (Di Trapani *et al* 2020). The results of the characterization have been subsequently used to tune a dedicated Geant4 simulation (Brombal *et al* 2022), which also included CdTe fluorescence effects and a realistic charge-sharing compensation mechanism. The output of the simulation allows to extrapolate the spectral response of the detector for any energy threshold and x-ray spectrum. The spectrum of the synchrotron beam was estimated by using the dedicated software Spectra (Tanaka 2021), while the effect of beam filtration was computed by using attenuation coefficients available in the XrayDB database (Elam *et al* 2002).

From the estimated x-ray spectrum and detector response, the energy response of the system was calculated for the threshold values, 26 and 33 keV, used in the acquisitions, as shown in figure 1(b). The low threshold (26 keV) was chosen to exclude the cadmium fluorescence, while the high threshold (33 keV) was set to maximize the iodine’s K-edge contrast (Di Trapani *et al* 2023). Additionally, the x-ray spectrum was shaped to be symmetric and centered on the second threshold, thus ensuring nearly equal statistics in the two energy bins.

The elements of the decomposition matrix were determined as the average of the tabulated mass attenuation and phase coefficients weighted over the spectrum and the spectral response of the detector:

\begin{eqnarray*}{\left(\frac{\mu }{\rho }\right)}_{i}^{L,H}=\frac{\int S(E){R}^{L,H}(E){\left(\frac{\mu }{\rho }\right)}_{i}(E){\mathrm{d}}E}{\int S(E){R}^{L,H}(E){\mathrm{d}}E};\ \ \ {\left(\frac{\delta }{\rho }\right)}_{i}=\frac{\int S(E)\left({R}^{L}(E)+{R}^{H}(E)\right){\left(\frac{\delta }{\rho }\right)}_{i}(E){\mathrm{d}}E}{\int S(E)\left({R}^{L}(E)+{R}^{H}(E)\right){\mathrm{d}}E}\end{eqnarray*}

where *S* is the polychromatic x-ray spectrum, *R* ^*L*,*H*^ are the detector energy response for the low and high energy bins, respectively, and *R* ^*L*^ + *R* ^*H*^ is the spectral response when only a single energy bin is used (non-spectral imaging).

### 2.4. Experimental setup and samples

The images were acquired at the SYRMEP beamline (Tromba *et al* 2010) of the Elettra Synchrotron facility (Trieste, Italy). The storage ring electron energy was 2.0 GeV and the current was 300 mA. X-ray radiation is produced through a bending magnet, generating a laminar beam with 7 mrad and 0.2 mrad of horizontal and vertical divergence, respectively. The beam dimension was defined with two pairs of motorized tungsten slits, resulting in a 3.8 mm high and 35 mm wide beam at the sample position. The desired polychromatic (pink) spectrum was obtained by filtering the white beam with 5 mm of aluminum and 120 mm of water, (see figure 1(b)). As a result, the flux at the detector when the two masks are aligned (i.e. top of the illumination curve) is in the order of 10^4^ photons/pixel/s, one order of magnitude lower than Pixirad’s linearity limit (Delogu *et al* 2016).

**Figure 3.**
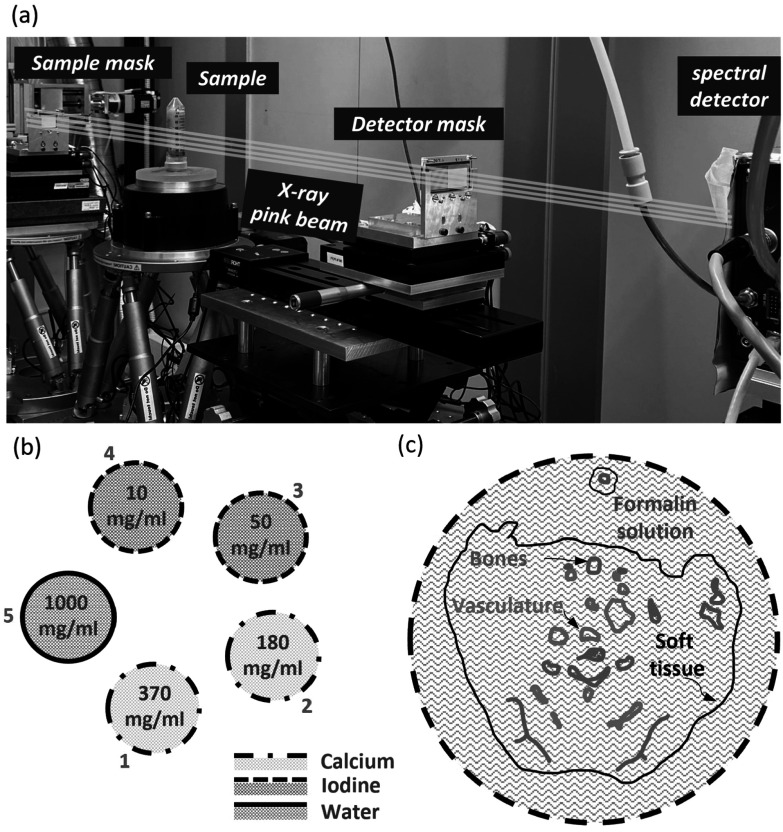
A photograph of the experimental setup (a) where the main components are outlined. In (b) and (c) the calibrated-cuvette sample and the mouse sample are sketched, respectively.

A photograph of the experimental setup showing the relevant instrumentation is shown in figure 3(a). The absorption masks featured a gold thickness of 250 *μ*m. The periods and apertures for the sample and detector masks were 116 *μ*m (period), 19 *μ*m (aperture) and 61 *μ*m (period), 10 *μ*m (aperture), respectively. The combination of masks’ periods and apertures corresponds to the so-called ‘skipped’ geometry, where every other detector pixel column is illuminated. This choice was made, among available options, to cope with the slight beam divergence and geometry of the SYRMEP beamline. The masks were mounted onto multi-axis positioning stages for alignment and stepping procedures. During the acquisition, 4 points on the illumination curve were acquired, corresponding to the left far slope (15%), both left and right half slopes (50%), and the top (100%). To ensure a complete sampling of the imaged object, 6 lateral displacements of the sample (i.e. dithering steps) were performed. The distances from the bending magnet source were 21.34 m for the sample mask, 21.84 m for the sample, 22.43 m for the detector mask, and 22.80 m for the detector.

Two samples were scanned. The first sample was made of 5 plastic cuvettes filled with solutions of known concentration. A sketch of the sample is shown in figure 3(b). Two cuvettes (number 1 and 2) were filled with CaCl_2_ solutions with densities of 370 mg ml^−1^ and 180 mg ml^−1^, two (number 3 and 4) were filled with iodine solutions with densities of 50 mg ml^−1^ and 10 mg ml^−1^, and the last one (number 5) contained pure distilled water. The concentrations of iodine were chosen to be similar to those commonly encountered in imaging applications making use of iodine-based contrast media. The second sample was an *ex vivo* 7 week old female athymic nude mouse (Charles River Laboratories, Wilmington, MA, USA), that received *post-mortem μ* Angiofil^®^ (Fumedica AG, Muri, Switzerland), a polymerizing iodine-based vascular contrast agent. The mouse was euthanized and afterward perfused with warm dPBS and contrast agent through the descending aorta. The euthanasia of the animal was performed according to the Helmholtz Zentrum Munich Animal Care and Use Committee guidelines. All relevant ethics and protocols for the study were in accordance with regulations of the government of Upper Bavaria. After euthanasia the *μ*Angiofil^®^ was left to polymerize for 1 h at room temperature. The sample was then shortly rinsed with dPBS, fixed in 4% paraformaldehyde, and stored at 4°C until *ex vivo μ*CT scanning for which it was inserted in a standard 50 ml Falcon tube. A sketch of a transversal slice of the mouse sample is shown in figure 3(c).

Tomographic images of the sample containing the calibrated cuvettes were acquired at an angular step of 0.5° over 180°. For the animal sample, which was larger than the detector field-of-view (FOV), the scan was carried out over 360° to perform the extended-FOV CT reconstruction (Wang 2002).

Spectral projection images were acquired with a chromatic photon-counting detector (Pixirad1-PixieIII). The detector features a 650 *μ*m thick CdTe sensor, a 62 *μ*m pixel size, and a sensitive area of 512 × 402 pixels, corresponding to 31.7 × 24.9 mm^2^ (Bellazzini *et al* 2015). The detector was operated in the two-color mode, through which incoming photons are recorded in low and high-energy bins. To ensure the best spectral performance and a small cross-talk between adjacent illuminated pixels, the charge-sharing compensation mode (NPISUM) was enabled during acquisition (Di Trapani *et al* 2020). The exposure time per each step of the acquisition was set to 1.2 s. Considering the number of dithering steps (6), illumination curve points (4), and projections (360 for the 180° scan and 720 for the 360° scan), the total exposure time was 2.9 h for the cuvette sample and 5.8 h for the mouse sample. From the number of detected photons, the total fluence on the mouse sample was estimated to be in the order of 3 × 10^12^ photons cm^−2^ corresponding to an entrance air kerma in the order of 2 Gy.

### 2.5. Image reconstruction and analysis

Raw projection images acquired at different positions of the illumination curve were processed via GPU-based Gaussian fitting to retrieve attenuation and differential phase signals (Przybylski *et al* 2017, Brombal *et al* 2023a). Integral phase projections were obtained from the differential phase via a Wiener-filter regularized phase-integration algorithm (Massimi *et al* 2020). The parallel beam filtered back projection was then used to reconstruct 3D tomographic volumes from both the attenuation and integrated-phase projections. The cuvette sample is reconstructed with a 59 × 59 × 59 *μ*m^3^ voxel (equal to the effective pixel size), as it does not contain high-resolution features. Conversely, the mouse sample was reconstructed with a 20 × 20 × 20 *μ*m^3^ voxel size (equal to the dithering step), to preserve high-resolution details. After reconstruction, material decomposition is applied following all three different algorithms outlined in section 2.1.

The standard spectral material decomposition in equation (4) uses low-energy and high-energy bin attenuation reconstructions as input. The attenuation/phase decomposition, described in equation (6), is applied on phase and attenuation reconstructions obtained by summing the raw data of low-energy and high-energy bins (i.e. considering the whole energy spectrum). The spectral/phase decomposition in equation (7) uses low-energy, high-energy and phase reconstructions.

Following material decomposition, the cuvette sample images were used to estimate the accuracy and sensitivity of each algorithm. Signal (i.e. density), noise, and signal-to-noise ratio (SNR) were measured for each cuvette within circular regions of interest, while uncertainties were computed by taking the standard deviation of the measured quantity across five consecutive slices. It is worth mentioning that, owing to the accurate spectral characterization, no calibration was needed, and the output images were directly produced in a mg/ml density scale.

The full spectral and material-decomposed datasets of both samples are publicly available at (Brombal 2023b)

## 3. Results

### 3.1. Test object

**Figure 4.**
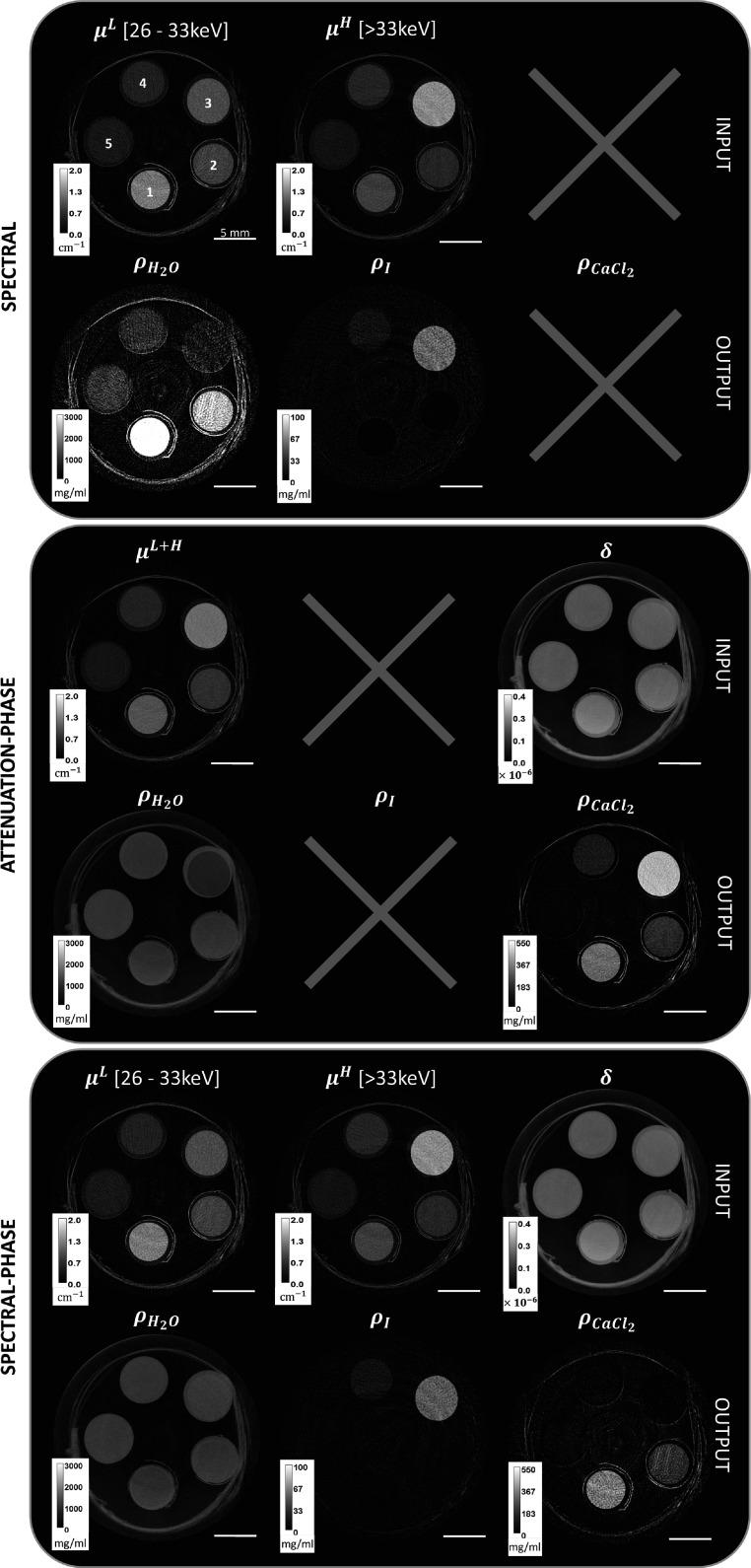
Input tomographic reconstructions and material decomposition outputs of the cuvette sample for each tested algorithm, namely spectral, attenuation/phase and spectral/phase.

Figure 4 shows the attenuation and phase CT reconstructions of the calibrated cuvette-sample, and their respective material decompositions obtained with spectral, attenuation/phase, and spectral/phase matrices as described in the previous section.

The first two rows show the conventional spectral imaging case. By comparing the two energy bins in the first row, it can be seen that the attenuation of the iodine-containing cuvettes (3 and 4) increases in the high energy bin due to the iodine K-edge, while attenuation reduces in calcium chloride and water-containing cuvettes (1, 2, and 5). Focusing on the decomposition in the second row, the iodine signal is found, as expected, only within cuvettes 3 and 4, and no contamination with water or calcium chloride is observed. On the other hand, the water map image shows strong signal contamination from the calcium chloride cuvettes, driving the density towards high values (∼4 g l^−1^ in cuvette number 1), and an underestimation of the water density (∼0.4 g l^−1^) in the highest-iodine concentration cuvette (number 3). Additionally, with respect to the input images, the output water map features higher image noise, boosted by matrix inversion.

The two central rows display the attenuation/phase decomposition. Compared to the non-spectral attenuation image (third row, first column), the phase image (third row, last column) shows a much smaller signal variation across the different cuvettes. This reflects the physics underlying equation (2), which demonstrates that the phase is solely dependent on the mass density. Given that the cuvette sample is composed of water solutions, the mass density in this specific case has a relatively small variation. Focusing on the decomposition maps (fourth row), it can be seen that calcium chloride is correctly quantified, yet a strong iodine signal contamination is observed in cuvettes 3 and 4. With respect to the conventional spectral decomposition, the water density image has a more uniform distribution except for the cuvette containing the highest concentration of iodine (number 3), where water density is underestimated. Overall, the attenuation/phase decomposition produces images with less noise with respect to the conventional spectral decomposition, mainly due to the higher SNR ratio of the input images.

The final two rows show the results of the spectral/phase decomposition, where the spectral attenuation input images are the same as those displayed in the first row, while the phase image is the same as reported in the third row. Focusing on the decomposition images (sixth row), all materials are correctly identified and no relevant signal contamination is observed. Additionally, owing to the low noise of the input phase channel, spectral/phase decomposition images show a significantly reduced level of noise compared to conventional spectral decomposition.

Table 1 summarizes the quantitative results obtained from the images displayed in figure 4. The measured mass densities in each cuvette for each algorithm, as well as their nominal values, are reported. It can be observed that, when present in the decomposition, the density of all materials is correctly reconstructed within three standard deviations. At the same time, the spectral/phase contrast decomposition is, on average, the closest to the nominal density: root mean square errors (rmse) computed from the last column of table 1 are of 1.1%, 1.9%, and 3.5% for water, iodine, and calcium chloride solutions, respectively. In the case of spectral decomposition rmse is of 1.3% for water and 4.8% for iodine, while in the attenuation/phase decomposition rmse is of 1.2% for water and 3.9% for calcium chloride.

**Table 1.** Quantitative results obtained from the images displayed in figure 4, comparing the three algorithms, namely spectral, attenuation/phase, and spectral/phase decompositions. The results are reported in terms of measured signal, noise, SNR, and relative error.

	Material [density (mg/ml)]	Signal (mg/ml)	Noise (mg/ml)	SNR	Δ (%)
Spectral	I [50]	51.1 ± 0.9	17.2 ± 1.1	2.97 ± 0.14	2.2
	I [10]	10.6 ± 0.2	12.8 ± 0.8	0.83 ± 0.07	6.3
	CaCl_2_ [370]	/	/	/	/
	CaCl_2_ [185]	/	/	/	/
	H_2_O [1000]	987 ± 17	739 ± 26	1.34 ± 0.04	−1.3


Attenuation/phase	I [50]	/	/	/	/
	I [10]	/	/	/	/
	CaCl_2_ [370]	350 ± 10	65.5 ± 2.4	5.35 ± 0.27	−5.4
	CaCl_2_ [185]	182 ± 4	59.8 ± 1.7	3.05 ± 0.08	−1.4
	H_2_O [1000]	988 ± 15	62.5 ± 2.1	15.8 ± 0.7	−1.2


Spectral/phase	I [50]	48.7 ± 0.7	12.6 ± 0.7	3.87 ± 0.18	−2.7
	I [10]	10.0 ± 0.2	9.3 ± 0.6	1.08 ± 0.08	0.1
	CaCl_2_ [370]	354 ± 9	115 ± 4	3.08 ± 0.13	−4.4
	CaCl_2_ [185]	181 ± 2	107 ± 4	1.69 ± 0.06	−2.4
	H_2_O [1000]	989 ± 14	82.3 ± 2.6	12.0 ± 0.5	−1.1

It is important to note that images of calcium chloride could have been obtained from the spectral algorithm, and images of iodine could have been obtained from the attenuation/phase algorithm, by swapping the used basis materials. However, this would have resulted in the loss of quantitative information about the material that was not included in the decomposition, in a similar way to that reported in table 1. Only the spectral/phase contrast decomposition allows for the simultaneous quantification of all the 3 material densities.

Examining SNR values, spectral/phase decomposition shows an improvement by a factor of 9 in the water image, and by a factor of 1.3 in iodine images compared to standard spectral decomposition. Conversely, compared to the attenuation/phase algorithm, spectral/phase decomposition shows a lower SNR, corresponding to a ∼25% decrease in the water image and a ∼45% reduction in the calcium chloride image. This can be readily explained by considering two factors: the different noise content of the input images and the different number of output decomposition materials between the two algorithms. These aspects will be further discussed in the following section.

### 3.2. Murine model

**Figure 5.**
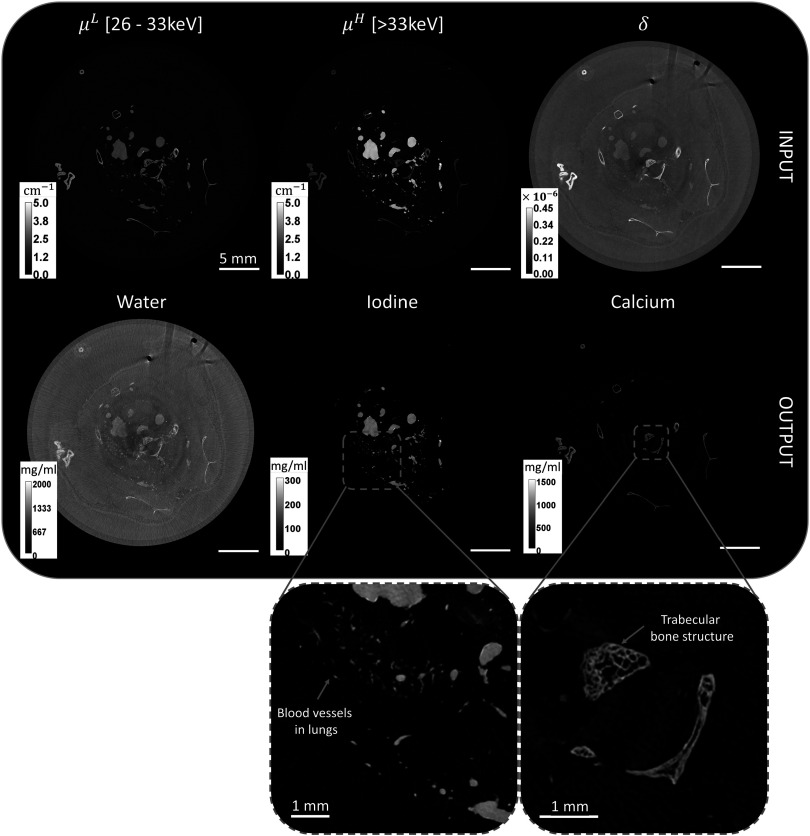
Input tomographic reconstructions and material decomposition output of the *post-mortem μ*Angiofil^®^-perfused mouse sample using spectral/phase algorithm. In the third row, the zoomed-in details of the iodine and calcium maps.

Figure 5 shows the reconstruction images of the *post-mortem μ*Angiofil^®^-perfused mouse sample. By focusing on the spectral attenuation images in the first row, *μ* ^*L*^ and *μ* ^*H*^, it is only possible to discern the signal of bones and iodine-perfused vasculature, while no soft tissue can be identified. On the contrary, in the phase input reconstruction, soft tissue structures such as the cutaneous and subcutaneous layers and internal organs are visible, despite the sample being soaked in formalin and immersed in a formalin background. The spectral/phase contrast decomposition into water (soft tissues), iodine (vasculature), and calcium (bone tissue) is shown in the second row of images. From calcium and iodine images, bone tissue and vasculature are clearly distinguishable, and no contamination signal is observed. The soft tissue and formalin background signals exclusively appear in the water channel, where a residual signal in correspondence to bones and blood vessels is expected as they are not composed of pure calcium and iodine, respectively. The zoomed-in details of the iodine and calcium maps in the third row highlight the capability of the system to capture small blood vessels (<50 *μ*m) as well as the fine trabecular structure of the bone.

**Figure 6.**
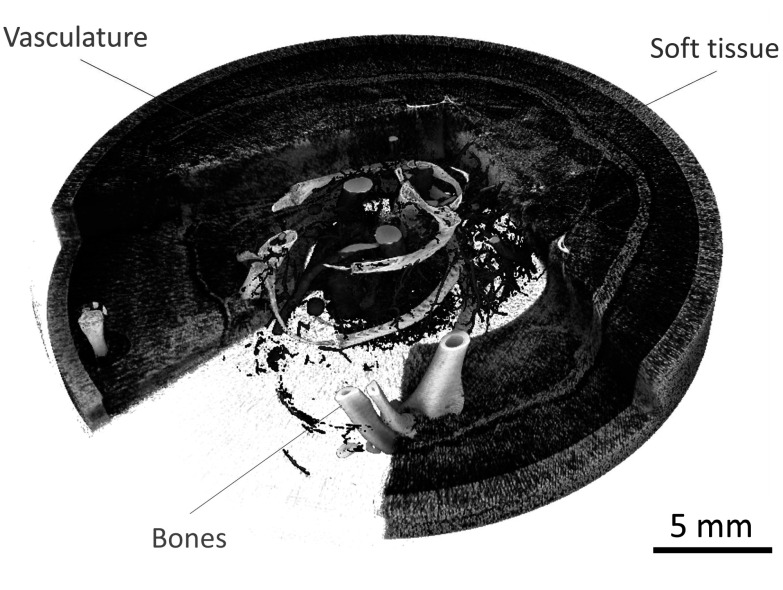
3D rendering of the *μ* Angiofil^®^-perfused mouse sample using the three decomposition channels, namely water (soft tissues, blue), iodine (vasculature, red), and calcium (bones, white).

A 3D rendering of the murine sample reconstruction after spectral phase contrast material decomposition is shown in figure 6. The water channel, displayed with the blue palette, is cut along axial and longitudinal planes to reveal the calcium (white palette) and iodine (red palette) channels. The rendering further demonstrates the simultaneous visualization soft tissues, bones, and vasculature.

## 4. Discussion and conclusions

The direct comparison of the three decomposition algorithms (figure 4) allows us to understand how the choice of different input images affects the output density maps. When aiming at contrast agent quantification, spectral attenuation information (*μ* ^*L*^, *μ* ^*H*^) is the most appropriate, as the contrast medium K-edge jump is specific to the attenuation coefficient. For this reason, when phase-channel information is added to the spectral attenuation channels, the SNR increase of the iodine map is modest with respect to the conventional spectral case (30%).

On the other hand, the differentiation of biological tissues (i.e. no K-edge materials) greatly benefits from the addition of the phase (*δ*) to the total attenuation (*μ* ^*L*+*H*^) channel. This is due to two main reasons. Firstly, *δ* maps have, especially for soft tissues, a generally higher SNR with respect to attenuation maps. A higher SNR input in the material decomposition algorithm results in higher SNR output images. Secondly, the presence in the decomposition matrix of terms with intrinsically different physical meanings guarantees linear independence across matrix rows, hence limiting the noise amplification due to matrix inversion (Di Trapani *et al* 2022).

The sensitivity to spectral and phase contrast is required to target a three-material decomposition image accounting for both the biological tissues and the contrast agent. As demonstrated in this work, high decomposition accuracy can be achieved on three materials relevant to biomedical CT imaging, namely water, iodine, and calcium. Root mean square differences from nominal density values smaller than 2% for water and iodine channels and smaller than 4% for the calcium channel (table 1) are found, while observing no contamination across different channels. At the same time, spectral/phase decomposition allows a high SNR in the soft tissue channels, as proven by the 9-fold increase in SNR on the water channel compared to the conventional spectral algorithm. The slight decrease of SNR in water and calcium channels observed when comparing spectral/phase and the attenuation/phase cases can be readily explained considering that, in the former, attenuation images with a lower SNR are given in input to the algorithm. Specifically, the two spectral attenuation images (*μ* ^*L*^, *μ* ^*H*^) used for the spectral/phase decomposition process exhibit a noise level that is 30% higher compared to the attenuation input image (*μ* ^*L*+*H*^) of the attenuation/phase algorithm. Additionally, the request for three output images instead of two (as in the attenuation/phase case) causes a noise amplification in the matrix inversion that is due to the intrinsic ill-conditioned nature of the spectral decomposition problem (Di Trapani *et al* 2022), which relates noise to the number of outputs. In this context, the possibility of using the energy-binned phase channels (*δ* ^*L*^, *δ* ^*H*^) combined with matrix inversion algorithms making use of data redundancy in a four-input/three-output scheme will be investigated in further studies.

The application of spectral phase contrast to the naked mouse reveals the potential of the technique on a biologically relevant sample (figure 5). The material decomposition process resulted in a clear separation of the vasculature (iodine map) and of the bones (calcium map). In addition, owing to the contribution of the phase channel, good visibility of the soft tissue formalin-fixed component has been retained (figure 6). It should be remarked that the high radiation dose levels (∼2 Gy) delivered during the scan are motivated by the fact that this is a proof-of-principle study, hence no dose reduction strategies have been implemented during the acquisition. For in-vivo applications, radiation dose reduction to levels of hundreds of mGy, commonly encountered in pre-clinical scanners (Boone *et al* 2004), can be achieved by optimizing the design of the masks, adopting more dose-efficient acquisition schemes, such as cycloidal tomography (Hagen and Vittoria 2020), or by reducing image noise through dedicated data-processing (Di Trapani *et al* 2022).

To the best of the authors’ knowledge, this is the first demonstration of a spectral phase-contrast CT on edge-illumination data. Moreover, compared to the work of Ji *et al* (2020), who first introduced the framework of spectral phase-contrast by making use of Talbot-Laue GI, this paper shows the first 3D result on an (*ex vivo*) entire animal sample at a reconstruction voxel size 4-times smaller (20 *μ*m versus ∼80 *μ*m). The presented approach can in principle be further translated to any phase-contrast imaging technique, including those based on wavefront marking, such as speckle imaging. At the same time, due to its achromaticity (Endrizzi *et al* 2014), i.e. tolerance to broad polychromatic x-ray spectra as required by spectral applications, edge-illumination is particularly suited for spectral phase-contrast imaging.

Although the present study makes use of synchrotron radiation, the translation of the setup and techniques herein presented on a laboratory setup involving a tungsten anode x-ray tube and targeting different contrast elements is straightforward provided that penumbra and related beam-hardening effects due to the beam divergence are considered. Specifically, a spectrum similar to the one presented in this paper can be obtained by operating an x-ray tube at a voltage of 50 kV by adding a filtration of 4 mm of aluminum.

It is the authors’ belief that the parallel diffusion of spectral detectors and laboratory-compatible phase-contrast techniques will inevitably lead to the integration of these two techniques in a range of applications. In this context, theoretical and experimental studies, such as the one reported in the present paper, have a potential impact on the design and operation of future scanners both in pre-clinical and clinical research.

## Data Availability

The data that support the findings of this study are openly available at the following URL/DOI:https://doi.org/10.15161/oar.it/143377.

## References

[pmbad3328bib1] Als-Nielsen J, McMorrow D (2011). Elements of Modern X-ray Physics.

[pmbad3328bib2] Alvarez R E, Macovski A (1976). Energy-selective reconstructions in x-ray computerised tomography. Phys. Med. Biol..

[pmbad3328bib3] Astolfo A, Haig I G, Bate D, Olivo A, Modregger P (2023). Increased material differentiation through multi-contrast x-ray imaging: a preliminary evaluation of potential applications to the detection of threat materials. Phys. Scr..

[pmbad3328bib4] Badea C T, Clark D P, Holbrook M, Srivastava M, Mowery Y, Ghaghada K (2019). Functional imaging of tumor vasculature using iodine and gadolinium-based nanoparticle contrast agents: a comparison of spectral micro-CT using energy integrating and photon counting detectors. Phys. Med. Biol..

[pmbad3328bib5] Bellazzini R, Brez A, Spandre G, Minuti M, Pinchera M, Delogu P, De Ruvo P, Vincenzi A (2015). PIXIE III: a very large area photon-counting CMOS pixel ASIC for sharp x-ray spectral imaging. J. Instrum..

[pmbad3328bib6] Boone J M, Velazquez O, Cherry S R (2004). Small-animal x-ray dose from micro-CT. Molecular Imaging.

[pmbad3328bib7] Braig E (2018). Direct quantitative material decomposition employing grating-based x-ray phase-contrast CT. Sci. Rep..

[pmbad3328bib8] Bravin A, Coan P, Suortti P (2012). X-ray phase-contrast imaging: from pre-clinical applications towards clinics. Phys. Med. Biol..

[pmbad3328bib9] Brombal L, Arfelli F, Menk R H, Rigon L, Brun F (2023a). Pepi lab: a flexible compact multi-modal setup for x-ray phase-contrast and spectral imaging. Sci. Rep..

[pmbad3328bib10] Brombal L, Rigon L, Arfelli F, Menk R, Brun F (2022). A geant4 tool for edge-illumination x-ray phase-contrast imaging. J. Instrum..

[pmbad3328bib50] Brombal L (2023b). Edge-illumination spectral phase-contrast tomography [Data set]. INFN Open Access Repository.

[pmbad3328bib11] Brun F (2020). Single-shot k-edge subtraction x-ray discrete computed tomography with a polychromatic source and the pixie-iii detector. Phys. Med. Biol..

[pmbad3328bib12] Buchanan I, Astolfo A, Endrizzi M, Bate D, Olivo A (2022). Reliable material characterization at low x-ray energy through the phase-attenuation duality. Appl. Phys. Lett..

[pmbad3328bib13] Delogu P, Oliva P, Bellazzini R, Brez A, De Ruvo P, Minuti M, Pinchera M, Spandre G, Vincenzi A (2016). Characterization of pixirad-1 photon counting detector for x-ray imaging. J. Instrum..

[pmbad3328bib14] Di Trapani V, Bravin A, Brun F, Dreossi D, Longo R, Mittone A, Rigon L, Delogu P (2020). Characterization of the acquisition modes implemented in pixirad-1/pixie-iii x-ray detector: effects of charge sharing correction on spectral resolution and image quality. Nucl. Instrum. Methods Phys. Res..

[pmbad3328bib15] Di Trapani V, Brombal L, Brun F (2022). Multi-material spectral photon-counting micro-ct with minimum residual decomposition and self-supervised deep denoising. Opt. Express.

[pmbad3328bib16] Di Trapani V, Oliva P, Arfelli F, Brombal L, Menk R H, Delogu P (2023). Development and validation of a simulation tool for k-edge subtraction imaging with polychromatic spectra and x-ray photon counting detectors. Nucl. Instrum. Methods Phys. Res..

[pmbad3328bib17] Elam W, Ravel B, Sieber J (2002). A new atomic database for x-ray spectroscopic calculations. Radiat. Phys. Chem..

[pmbad3328bib18] Endrizzi M (2018). X-ray phase-contrast imaging. Nucl. Instrum. Methods Phys. Res..

[pmbad3328bib19] Endrizzi M, Diemoz P C, Millard T P, Louise Jones J, Speller R D, Robinson I K, Olivo A (2014). Hard x-ray dark-field imaging with incoherent sample illumination. Appl. Phys. Lett..

[pmbad3328bib20] Evans R D (1955). The Atomic Nucleus.

[pmbad3328bib21] Faby S, Kuchenbecker S, Sawall S, Simons D, Schlemmer H-P, Lell M, Kachelrieß M (2015). Performance of today’s dual energy CT and future multi energy CT in virtual non-contrast imaging and in iodine quantification: a simulation study. Med. Phys..

[pmbad3328bib22] Flohr T, Petersilka M, Henning A, Ulzheimer S, Ferda J, Schmidt B (2020). Photon-counting CT review. Phys. Med..

[pmbad3328bib23] Frank M (2022). Dark-field chest x-ray imaging for the assessment of covid-19-pneumonia. Commun. Med..

[pmbad3328bib24] Gureyev T (2019). Propagation-based x-ray phase-contrast tomography of mastectomy samples using synchrotron radiation. Med. Phys..

[pmbad3328bib25] Hagen C K, Vittoria F A, i Morgó O R, Endrizzi M, Olivo A (2020). Cycloidal computed tomography. Phys. Rev. Appl..

[pmbad3328bib26] Havariyoun G (2019). A compact system for intraoperative specimen imaging based on edge illumination x-ray phase contrast. Phys. Med. Biol..

[pmbad3328bib27] Hsieh S S, Leng S, Rajendran K, Tao S, McCollough C H (2020). Photon counting CT: clinical applications and future developments. IEEE Trans. Radiat. Plasma Med. Sci..

[pmbad3328bib28] Ji X, Zhang R, Li K, Chen G-H (2020). Dual energy differential phase contrast ct (de-dpc-ct) imaging. IEEE Trans. Med. Imaging.

[pmbad3328bib29] Liu X, Yu L, Primak A N, McCollough C H (2009). Quantitative imaging of element composition and mass fraction using dual-energy CT: three-material decomposition. Med. Phys..

[pmbad3328bib30] Longo R (2019). Advancements towards the implementation of clinical phase-contrast breast computed tomography at elettra. J. Synchrotron Radiat..

[pmbad3328bib31] Massimi L, Buchanan I, Astolfo A, Endrizzi M, Olivo A (2020). Fast, non-iterative algorithm for quantitative integration of x-ray differential phase-contrast images. Opt. Express.

[pmbad3328bib32] Massimi L (2021). Volumetric high-resolution x-ray phase-contrast virtual histology of breast specimens with a compact laboratory system. IEEE Trans. Med. Imaging.

[pmbad3328bib33] Mechlem K, Sellerer T, Viermetz M, Herzen J, Pfeiffer F (2019). Spectral differential phase contrast x-ray radiography. IEEE Trans. Med. Imaging.

[pmbad3328bib34] Navarrete-León C (2023). X-ray phase-contrast microtomography of soft tissues using a compact laboratory system with two-directional sensitivity. Optica.

[pmbad3328bib35] Olivo A, Speller R (2007). A coded-aperture technique allowing x-ray phase contrast imaging with conventional sources. Appl. Phys. Lett..

[pmbad3328bib36] Paakkari P, Inkinen S I, Honkanen M K M, Prakash M, Shaikh R, Nieminen M T, Grinstaff M W, Mäkelä J T A, Töyräs J, Honkanen J T J (2021). Quantitative dual contrast photon-counting computed tomography for assessment of articular cartilage health. Sci. Rep..

[pmbad3328bib37] Partridge T, Astolfo A, Shankar S, Vittoria F, Endrizzi M, Arridge S, Riley-Smith T, Haig I, Bate D, Olivo A (2022). Enhanced detection of threat materials by dark-field x-ray imaging combined with deep neural networks. Nat. Commun..

[pmbad3328bib38] Polikarpov M (2023). Towards virtual histology with x-ray grating interferometry. Sci. Rep..

[pmbad3328bib39] Przybylski A, Thiel B, Keller-Findeisen J, Stock B, Bates M (2017). Gpufit: an open-source toolkit for gpu-accelerated curve fitting. Sci. Rep..

[pmbad3328bib40] Quenot L, Bohic S, Brun E (2022). X-ray phase contrast imaging from synchrotron to conventional sources: a review of the existing techniques for biological applications. Appl. Sci..

[pmbad3328bib41] Rawlik M (2023). Increased dose efficiency of breast CT with grating interferometry. Optica.

[pmbad3328bib42] Roessl E, Proksa R (2007). K-edge imaging in x-ray computed tomography using multi-bin photon counting detectors. Phys. Med. Biol..

[pmbad3328bib43] Schaff F, Morgan K S, Pollock J A, Croton L C, Hooper S B, Kitchen M J (2020). Material decomposition using spectral propagation-based phase-contrast x-ray imaging. IEEE Trans. Med. Imaging.

[pmbad3328bib44] Schlomka J (2008). Experimental feasibility of multi-energy photon-counting k-edge imaging in pre-clinical computed tomography. Phys. Med. Biol..

[pmbad3328bib45] Sukovle P, Clinthorne N (1999). Basis material decomposition using triple-energy x-ray computed tomography, IMTC/99.

[pmbad3328bib46] Tanaka T (2021). Major upgrade of the synchrotron radiation calculation code spectra. J. Synchrotron Radiat..

[pmbad3328bib47] Tromba G (2010). The syrmep beamline of elettra: clinical mammography and bio-medical applications.

[pmbad3328bib48] Wang G (2002). X-ray micro-CT with a displaced detector array. Med. Phys..

[pmbad3328bib49] Willemink M J, Persson M, Pourmorteza A, Pelc N J, Fleischmann D (2018). Photon-counting CT: technical principles and clinical prospects. Radiology.

